# Patients' Perspectives of Accessibility and Digital Delivery of Factual Content Provided by Official Medical and Surgical Specialty Society Websites: A Qualitative Assessment

**DOI:** 10.2196/ijmr.3963

**Published:** 2015-03-27

**Authors:** Darren Ow, David Wetherell, Nathan Papa, Damien Bolton, Nathan Lawrentschuk

**Affiliations:** ^1^Austin HospitalDepartment of SurgeryUniversity of MelbourneMelbourneAustralia; ^2^Olivia Newton-John Cancer Research InstituteAustin HospitalMelbourneAustralia; ^3^Peter MacCallum Cancer CentreDepartment of Surgical OncologyMelbourneAustralia

**Keywords:** Internet, online health information, patient education, social media, Web science

## Abstract

**Background:**

Health care websites provide a valuable resource of health information to online consumers, especially patients. Official surgical and medical society websites should be a reliable first point of contact.

**Objective:**

The primary aim of this study was to quantitatively assess medical and surgical society websites for content and highlight the essential features required for a high-quality, user-friendly society website.

**Methods:**

Twenty specialty association websites from each of the regions, Australia, UK, Canada, Europe, and the USA were selected for a total of 100 websites. Medical and surgical specialities were consistent across each region. Each website was systematically and critically analysed for content and usability.

**Results:**

The average points scored per website was 3.2 out of 10. Of the total (N=100) websites, 12 scored at least 7 out of 10 points and 2 scored 9 out of 10. As well, 35% (35.0/100) of the websites had an information tab for patients on their respective homepages while 38% (38.0/100) had download access to patient information. A minority of the websites included different forms of multimedia such as pictures and diagrams (24.0/100, 24%) and videos (18.0/100, 18%).

**Conclusions:**

We found that most society websites did not meet an adequate standard for delivery of information. Half of the websites were not patient accessible, with the primary focus being for health professionals. As well, most required logins for information access. Specialty health care societies should create patient-friendly websites that would be beneficial to all online consumers.

## Introduction

Advances in modern technology and communication have resulted in a new digital age where the Internet is an important source of information for health professionals and consumers. The number of health care providers and consumers gaining access to this information is expanding [1]. The Pew Internet and American Life Project reported that 80% of adults in the United States of America (USA) sought health information via search engines (Google, Bing, or Yahoo) [2]. Many specialities have embraced this technological advance. For example, in recent years, urology has been open to integrating the Internet and social media as a new communication platform [3]. Although social media has been around for the past decade and is widely used in other spheres, it has not been utilised as much in the health community, and only a small number of health faculties engage in social media [4]. Additionally, there is a wide disparity in the quality of health information on the Internet and not all accredited and quality information is readily accessible for the online consumer.

Official surgical and medical society websites are a valuable resource of health information for professionals and patients. They allow for the centralisation of information in a user-friendly and accessible format. Within this niche of websites, the quality of information remains variable. Our primary aim was to systematically assess these websites for content, quality, and delivery of health information. We also intended to highlight the key features required for a high-quality society website.

## Methods

Medical and surgical society websites (N=100) were systematically reviewed in September 2012. Websites (n=20) from each of the regions (Australia, Canada, Europe, United Kingdom, and USA) were collected from various medical (cardiology, endocrinology, gastroenterology, haematology, infectious disease, nephrology, neurology, oncology, respiratory and rheumatology) and surgical (cardiothoracic, general surgery, maxillofacial, neurosurgery, otolaryngology, paediatric surgery, plastics, urology and vascular) specialties. A Google search was conducted to identify the websites using the keywords medical or surgical specialty name, society or association, and country. All selected websites were in English.

**Figure 1:**
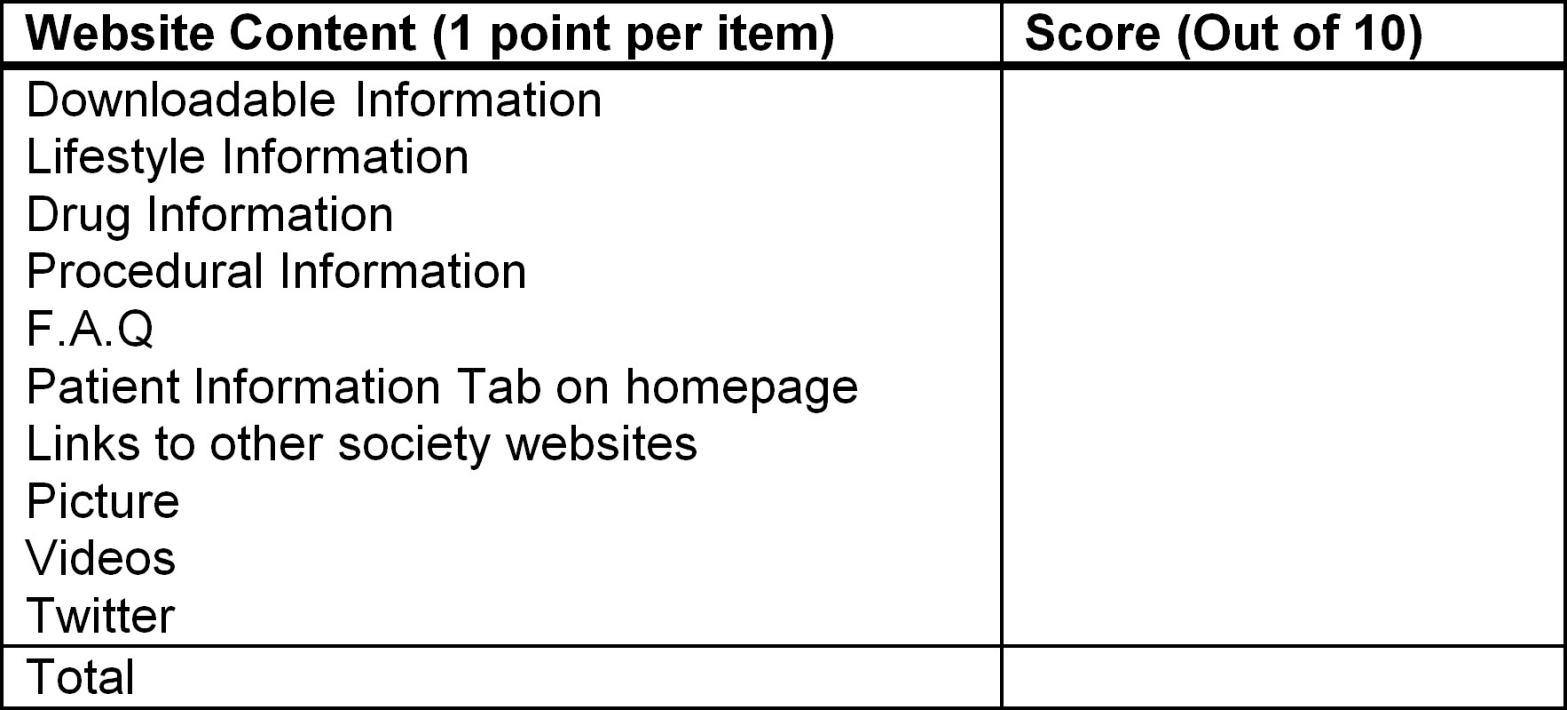
Website content and usability scoring sheet.

Rafe et al constructed a qualitative framework to assess hospital and other medical websites that focused on 7 key metrics, which are (1) content, (2) design, (3) organization, (4) user-friendliness, (5) performance, (6) service, and (7) technical quality [5]. Using this framework of content and user-friendliness metrics, we constructed a simple 10-point quality appraisal tool. This tool was designed to assess the usability of websites for health professionals, patients, and other online consumers. The 10 points included information about procedures, drugs, and lifestyle interventions, a frequently asked questions (FAQs) page, pictures and diagrams, video attachments, social media links (ie, Twitter), presence of a patient information tab on the homepage, ability to easily download information, and inclusion of relevant website links (Figure 1).

**Figure 2:**
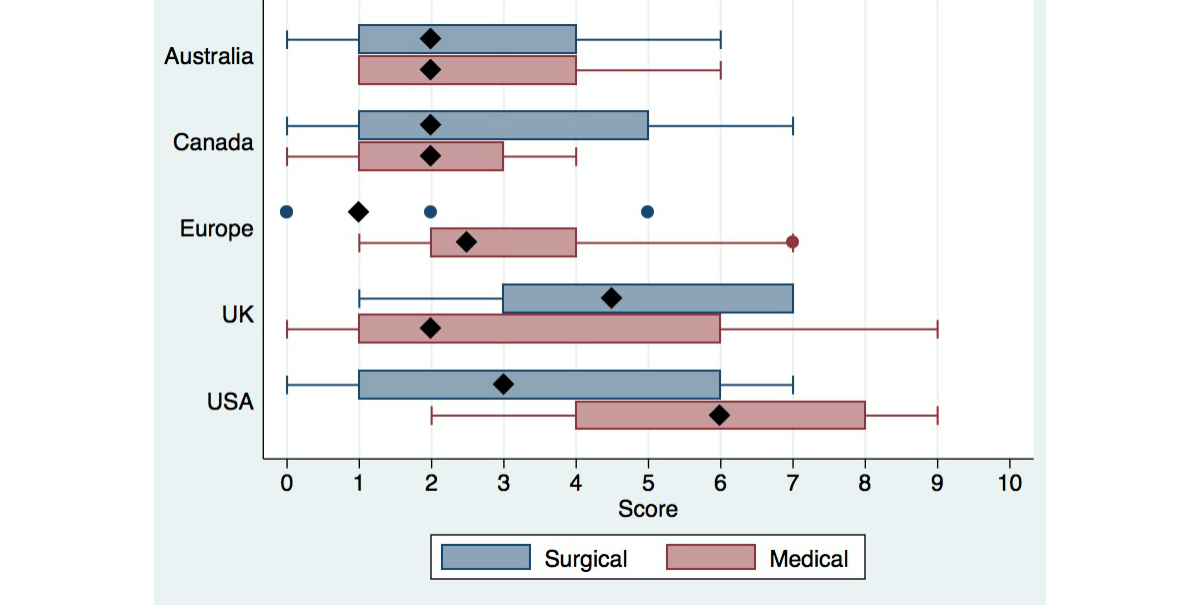
Webscores based on country and specialty.

The variables were evaluated for statistically significant differences between regions and specialties using the non-parametric Kruskal-Wallis equality of population test. Statistical significance was set at *P*<.05. The scores were graphed using a box and whisker plot (Figure 2). Statistical analysis was performed using Stata v.12.0 SE (College Station, Texas).

## Results

**Figure 3:**
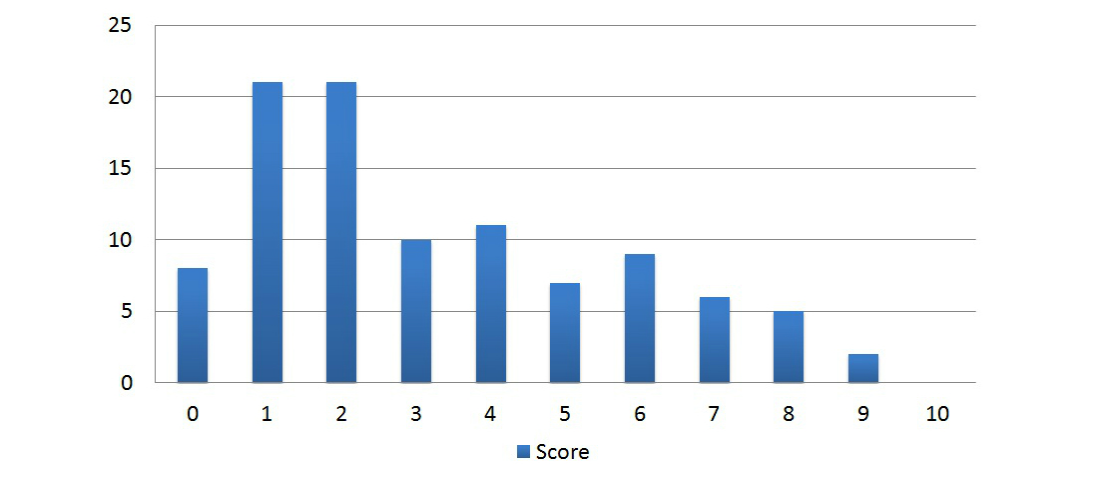
Total website scores based on points.

A total of 100 health care society websites were selected in this study with an equal number of medical and surgical specialties. The mean points scored per website was 3.2 (range 0-9, SD 2.4) and the median points per site was 2.5. Of the websites assessed, 12 scored at least 7 points, 2 scored 9 points, and 28 scored between 4-6 points out of a total of 10. The remaining 60% (60.0/100) of websites scored 3 or less points with 9 scoring 0 points (Figure 3).

Among the 5 regions, USA health care websites had the highest mean website score of 4.6, followed by UK websites with a mean score of 4.0. Australian and Canadian health care society websites scored 2.7 and 2.6, respectively, while the European health care society websites scored a mean of 2.2 points. Statistical testing revealed a significant difference between the regions (*P*=.01). When comparing medical and surgical specialties, surgical specialties had a mean score of 2.9 points per website, while medical specialties had a mean score of 3.4 points. These scores were not statistically significant.

Of the 100 society websites, 35 (14 medical, 21 surgical) displayed a patient information tab on their main webpage. Links to other health care related websites were displayed on 60 websites. A minority of websites included different forms of multimedia such as pictures and diagrams (24.0/100, 24%) and videos (18.0/100, 18%). As well, 38 websites (15 medical, 23 surgical) had information that was easily downloadable without any login requirements, 27 (15 medical, 12 surgical) had lifestyle intervention information, 21 (14 medical, 7 surgical) had drug information, 36 (14 medical, 22 surgical) had procedural information, and 7 had a FAQs page. Out of 100 associations, 61 (61.0/ 61%) had active Twitter accounts and of these, 52 (85.0/100, 85%) had direct links to these accounts from their websites.

## Discussion

### Principal Findings

Our website quality appraisal tool was initially designed to assess the accessibility of health care society websites from a patient's perspective. Almost all medical and surgical societies have established official websites. These websites should be the first point of reference for online health information relevant to a particular condition covered by that specialty. However, these websites achieved a wide range of scores when the quality appraisal tool was applied. Only a few health care society websites managed to score 7 or more points while most failed to display adequate content and usability, thus making it difficult for patients and health care professionals to use the websites as a reliable information source.

We found that about one third of these official websites had patient information tabs in their homepage with the incentive being for patients to gain a better understanding of their own medical condition or about the specialty. A number of websites were designed with pictures (n=24) and videos (n=18) attached to the relevant conditions discussed [6].

Other forms of multimedia such as podcasts and power point lectures were provided more for health care professionals. In terms of drug information being displayed on websites, medical societies had higher scores than surgical societies, while the reverse was found with respect to procedural information. This might be explained by the fact that medical specialties make more use of treatments with medication rather than surgical procedures.

Interestingly we observed that website scores varied for each region. Both the USA and UK society websites had higher scores than other regions. Clearly, these websites showed a more user-friendly approach towards patients than Australian, Canadian, and European society websites. European health care society websites scored the lowest among the other regions, explained by the paucity of the available written and multimedia information. However, this may also be due to the possibility of alternative websites in other languages.

One possible reason the USA and UK websites scored highly is that at the time of review, about 80% (80.0/100) of USA and UK health care society websites had links to official Twitter accounts, while only 20% (20.0/100) of the Australian and Canadian society websites had an official Twitter account link. Some health care societies have used Twitter as a platform to engage with the public community or even with health care professionals [7]. Twitter has been utilised at many conferences to engage in clinical discussions and to further the communication outside of the conference [8,9]. Despite the exponential increase of Twitter use in the medical field over the past few years, these forms of social media are still not widely accepted in certain countries [10].

There is a proliferation of medical information websites on the Internet, most of uncertain quality. As an official society website, the health information provided therein is considered reliable and accurate. However, websites requiring a login to access online information or other features may deter patients from exploring the website any further. A possible explanation for restricted access could be that the majority of the websites were designed for health care professionals rather than patients. It would be ideal if this information was more widely disseminated to all health consumers [11]. Free access to online information could potentially be an alternative method to deliver information and improve communications between health care professionals and patients, thus narrowing the gap to health care services [12].

### Limitations

A limitation to the study was that some of the medical societies had more than one official website and therefore, there was a lack of centralisation of information. Therefore, it is our opinion that society-association websites should be unified, although this may not be feasible in certain circumstances where websites have already been established.

The appraisal tool used in this study was designed towards a patient-focused evaluation rather than health care professional. Therefore, other potential key roles of society websites that were designed for health care have not been evaluated. Also, this study only included English language websites and may be biased towards regions where English is not the first language.

The presence of Twitter links to each health care society websites were reviewed, however, the level of Twitter activity was not assessed. Twitter activity could potentially be a better indicator of engagement with online consumers.

### Conclusions

In this era, the population of online users seeking health information has risen, and health care societies should try to adapt and make the transition to developing a higher standard of website. This will engage and encourage patients to participate in their health management rather than being a passive recipient of health care. Creating good quality websites by using a patient-friendly framework would be beneficial to all online consumers. However, most society websites were created specifically for health care professionals rather than patients. Furthermore, these health care society websites were often not user-friendly for the patient with some requiring a login for information access. Although specialty health care societies-associations’ websites have been established for health care professionals, these websites need to become more education-focused for patients if they are to be the lead voice in their area of practice, and to improve their craft’s profile within the wider community.

## Abbreviations

FAQs: frequently asked questions
